# The complete chloroplast genome sequence of *Cenchrus purpureus*

**DOI:** 10.1080/23802359.2018.1536451

**Published:** 2018-11-25

**Authors:** Yang Wu, Hui Zhou

**Affiliations:** a Department of Biotechnology, Fujian Vocational College of Agriculture, Fuzhou, China;; b Sugarcane Research Institute, Guangxi Academy of Agricultural Sciences, Nanning, China;; c Guangxi Collaborative Center for Sugarcane and Cane Sugar Industries, Nanning, China

**Keywords:** Chloroplast, *Cenchrus purpureus*, genome sequence

## Abstract

The complete chloroplast genome sequence of *Cenchrus Purpureus*, important silage in China, is presented in this article. The total genome size is 138,199 bp, containing a large single copy (LSC) region (81,161 bp) and a small single copy region (12,386 bp) which were separated by two inverted repeat (IRs) regions (22,326 bp). The overall GC contents of the plastid genome were 38.6%. In total, 136 unique genes were annotated and they were consisted of 87 protein-coding genes, 41 tRNA genes, and 8 rRNA genes. Twenty-four genes duplicated in the LSC and IR regions. Eighteen genes contained one or two introns.

*Cenchrus purpureus* Schum., formerly *Pennisetum purpureum* (Chemisquy et al. 2010), originated in the subtropical Africa, is widely known as Elephant Grass, Napier Grass, or King Grass (Rueda et al. 2016). This giant grass has high biomass yield, and aroused the interest of many researchers for serving as a fodder for animal feeding (Valle et al. 2009; Chen et al. 2010), a wood substitute for the paper-mill industry (Madakadze et al. 2010), and a charcoal substitute for bioenergy production by direct biomass combustion (Samson et al. 2005; Morais et al. 2009).

The complete chloroplast genome of *C. Purpureus* was sequenced by MiSeq desktop sequencer of Illumina (San Diego, CA), assembled into the complete chloroplast genome by MITObim v1.8 (Hahn et al. 2013) with the reference chloroplast genome of *C. americanus*, annotated by Dual Organellar GenoMe Annotator (DOGMA) (Wyman et al. 2004), and submitted to GenBank with the accession number of MF594682. The chloroplast DNA was extracted from a single individual *C. Purpureus* growing in germplasm garden of Sugarcane Research Institute, Guangxi Academy of Agricultural Sciences, Nanning, China (22°51′27″N 108°15′25″E). DNA sample of *C. Purpureus* were stored in the laboratory of Sugarcane Research Institute, Guangxi Academy of Agricultural Sciences, Nanning, China.

The total chloroplast genome size of *C. Purpureus* is 138,199 bp, containing a large single copy (LSC) region (81,161 bp) and a small single copy region (12,386 bp), which were separated by two inverted repeat (IRs) regions (22,326 bp). The overall GC contents of the plastid genome were 38.6%. In total, 136 unique genes were annotated, including 87 protein-coding genes, 8 rRNA genes, and 41 tRNA genes. There are 24 genes duplicated in the LSC and IR regions, including 9 protein-coding genes, 4 rRNA genes, and 11 tRNA genes. The protein-coding genes, rRNA genes, and tRNA genes account for 64.0%, 30.1%, and 5.9% of all annotated genes, respectively. Eighteen genes contained one or two introns, including the protein-coding genes, *atpF*, *ndhA*, *ndhB*, *petB*, *rpl2*, *rps12*, *rps16*, and *ycf3*. The Maximum Likelihood phylogenetic tree was generated using RAxML (Stamatakis 2014) based on the complete chloroplast genome of *C. Purpureus* and seven other species from the family Poaceae. The phylogenetic tree showed that *C. Purpureus* was closely related to *C. americanus* (Figure 1). This published *C. Purpureus* chloroplast genome will provide useful information for phylogenetic and evolutionary studies in *Cenchrus* and Poaceae.

**Figure 1.**
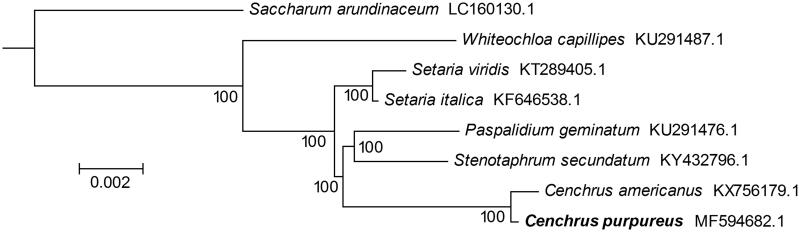
Maximum Likelihood phylogenetic tree generated byRAxMLbased on complete chloroplast genome sequences of eight species from the family Poaceae using S.arundinaceum as an outgroup. Numbers on branches are bootstrap support values based on 10,000 iterations.
